# Anti-Atherosclerotic Properties of *Aronia melanocarpa* Extracts Influenced by Their Chemical Composition Associated with the Ripening Stage of the Berries

**DOI:** 10.3390/ijms25084145

**Published:** 2024-04-09

**Authors:** Agnieszka Zielińska, Dorota Bryk, Katarzyna Paradowska, Paweł Siudem, Iwona Wawer, Małgorzata Wrzosek

**Affiliations:** 1 Department of Organic and Physical Chemistry, Faculty of Pharmacy, Medical University of Warsaw, Banacha 1, 02-097 Warsaw, Poland; agnieszka.zielinska@wum.edu.pl (A.Z.); katarzyna.paradowska@wum.edu.pl (K.P.);; 2 Department of Biochemistry and Pharmacogenomics, Faculty of Pharmacy, Medical University of Warsaw, Banacha 1, 02-097 Warsaw, Poland; dorota.bryk@wum.edu.pl; 3 Department of Herbology, State University of Applied Sciences, Rynek 1, 38-400 Krosno, Poland; profwawer@gmail.com; 4 Centre for Preclinical Research, Medical University of Warsaw, Banacha 1b, 02-097 Warsaw, Poland

**Keywords:** *Aronia melanocarpa*, anthocyanins, chlorogenic acids, inflammation, adhesion molecules, endothelial cells

## Abstract

The high content of bioactive compounds in *Aronia melanocarpa* fruit offers health benefits. In this study, the anti-atherosclerotic effect of Aronia extracts was assessed. The impact on the level of adhesion molecules and the inflammatory response of human umbilical vein endothelial cells (HUVECs) was shown in relation to the chemical composition and the stage of ripening of the fruits. Samples were collected between May (green, unripe) and October (red, overripe) on two farms in Poland, which differed in climate. The content of chlorogenic acids, anthocyanins, and carbohydrates in the extracts was determined using HPLC-DAD/RI. The surface expression of ICAM-1 and VCAM-1 in HUVECs was determined by flow cytometry. The mRNA levels of *VCAM-1*, *ICAM-1*, *IL-6*, and *MCP-1* were assessed using the quantitative real-time PCR method. The farms’ geographical location was associated with the quantity of active compounds in berries and their anti-atherosclerotic properties. Confirmed activity for green fruits was linked to their high chlorogenic acid content.

## 1. Introduction

The *Aronia melanocarpa* (Michx.) Ell., also known as black chokeberry or Aronia, is a shrub native to the United States and belongs to the *Rosaceae* family. In the first half of the 20th century, chokeberry varieties were introduced to the Soviet Union and other European countries [1]. Currently, its berries are used mainly to produce juices, jams, and wines, as well as plant remedies and natural dye (powdered extract) [2].

The *A. melanocarpa* fruit contains a significantly higher content of polyphenolic compounds than other dark-colored berries—on average, about 7000 mg/100 g of dry matter (DM). For comparison, the total polyphenol content varied from 916 to 2310 mg/100 g DM in blackberry, raspberry, and strawberry fruits [3]. Polyphenols, especially anthocyanins, proanthocyanidins, and phenolic acids (chlorogenic and neochlorogenic) are the largest group of biologically active constituents of *A. melanocarpa.* Chokeberry also contains quercetin derivatives, triterpenes, vitamins, tannins, and mineral salts [2,4,5]. In addition, the fruits of *A. melanocarpa* have an interesting carbohydrate profile. They contain significantly more sorbitol than glucose and fructose, with a negligible amount of sucrose [6]. The concentration of sorbitol is the highest among the series of fruits and berries; therefore, its use as a characteristic component in Aronia juices was postulated to detect its falsification with other juices, such as pear, apple, or black currant berries [7].

Anthocyanins constitute approximately 25% of all phenolic components of Aronia berries [8]. They are represented by cyanidin glycosides, mainly cyanidin-3-O-galactoside and cyanidin-3-O-arabinoside, and in much smaller amounts, cyanidin-3-O-glucoside and cyanidin-3-O-xyloside [9,10]. Anthocyanins contained in the diet are subject to complex metabolism. After being partially absorbed in the stomach and then in the small intestine, they appear in blood and urine in various forms, including intact, methylated, glucuronidated, and sulfoconjugated [11]. Unabsorbed anthocyanins are extensively metabolized by the intestinal microbiota. In vitro studies show that bacterial metabolism involves the cleavage of glycosidic linkages with the breakdown of the anthocyanidin heterocycle and degradation into phloroglucinol derivatives and benzoic acids. In addition, anthocyanins can stimulate the growth of beneficial bacteria, such as *Bifidobacterium* spp. and *Lactobacillus-Enterococcus* spp., and have positive effects on the gut microbiota [11]. Besides intact anthocyanins, primary human metabolites include simple phenolic acids like 4-hydroxybenzoic acid, protocatechuic acid, gallic acid, vanillic acid, and syringic acid [12]. After consuming chokeberry extracts, cyanidin 3-galactoside constitutes the majority of anthocyanins detected in human urine and serum samples (55.4% and 66.0%, respectively) [13]. Anthocyanins can benefit health through their documented antioxidant and anti-inflammatory effects [10]. The underlying mechanisms of their action are rather complex, i.e., they have an impact on intracellular signal transduction pathways, gene expression, and cellular functions [14].

Chokeberry contains an essential group of compounds known as phenolic acids, which includes chlorogenic (CGA, 3-O-caffeoylquinic acid) and neochlorogenic (nCGA, 5-O-caffeoylquinic acid) acids, both present in significant quantities. CGA is found in approximately 300 mg/100 g DW, while nCGA is present in 290 mg/100 g DW [7]. Other acids found in chokeberry include cryptochlorogenic, p-coumaric, caffeic, vanillic, ferulic, salicylic, syringic, and ellagic acids [7,15]. Among these, chlorogenic acid is the most extensively studied. When consumed, approximately one-third is absorbed in the stomach, but the absorption partially depends on hydrolysis. After hydrolysis, CGA is further conjugated with sulfate or glucuronic acid and then absorbed in the intestine, mostly after being metabolized by gut microbiota. Studies on rats have shown that CGA is metabolized into 5-O-feruloyl quinic acid, caffeic acid, ferulic acid, dihydrocaffeic acid, dihydroferulic acid, and 3-(3-hydroxyphenyl) propionic acid. The bioavailability of CGA depends on the form in which it is consumed (liquid or solid) and other products taken simultaneously [16]. The bioactivity of chlorogenic acids (CGAs) is associated with antioxidant and anti-inflammatory properties. In vitro and in vivo data indicate that CGAs can alleviate oxidative stress in various diseases [17,18]. Previous studies have shown the molecular mechanisms of action of nCGA, which lowered the production of key inflammatory markers, such as tumor necrosis factor-alpha (TNF-α), interleukin-6 (IL-6), and nitric oxide (NO), and further reduced the expression of inducible nitric oxide synthase (iNOS) and cyclooxygenase-2 (COX2) [19].

The high polyphenol content of chokeberry has piqued the interest of researchers searching for health-promoting applications beyond its use as a food product. Black chokeberry extracts have been shown, in both in vitro and in vivo studies, to exhibit anti-inflammatory, antioxidant, cardioprotective, immunomodulatory, gastroprotective, antidiabetic, hepatoprotective, antibacterial, antiviral, and antitumor activities [2,20,21]. Consumption of chokeberry fruit is recommended to prevent metabolic diseases, mainly due to the confirmed beneficial effect on lipid profile, plasma glucose concentrations, and blood pressure. Additionally, it shows potential as an anti-obesity food supplement due to its inhibition of pancreatic amylase and lipase [22]. Studies in animals and humans have demonstrated the impact of chokeberry on lowering lipid levels [23,24]. In addition, chokeberry reduced blood pressure, oxidative stress, and inflammation marker levels in patients with hypertension [25,26]. Antiplatelet and vasoprotective properties were also observed in porcine coronary arteries [27,28], as well as the protection of endothelial cells against angiotensin II-induced oxidative stress [27].

Inflammation in the endothelium induces the production of inflammatory factors and the adhesion of monocytes, which are crucial processes in the onset of atherosclerosis [28,29]. Monocytes and lymphocytes dominate in early atherosclerotic plaque. Intercellular adhesion molecule-1 (ICAM-1) and vascular cell adhesion molecule-1 (VCAM-1) are up-regulated in endothelial cells in response to cytokines, such as tumor necrosis factor-α (TNFα) and interleukin 6 (IL-6), chemokines, and growth factors [30,31]. This promotes the adhesion of the circulating monocytes, neutrophils, lymphocytes, and macrophages to the vascular cells. Monocyte chemoattractant protein-1 (MCP-1) is vital in directing monocytes/macrophages into the atherosclerotic lesions and promoting plaque progression [32,33]. Thus, medications or natural substances that suppress the expression of inflammatory factors and adhesion molecules may constitute an essential element in preventing or treating atherosclerotic diseases [34].

In this study, the anti-atherosclerotic effect of Aronia fruit extracts was carried out by measuring the expression of ICAM-1 and VCAM-1, IL-6, and MCP-1 in human umbilical vein endothelial cells (HUVECs). The *A. melanocarpa* extracts were obtained from fruits harvested from two plantations in Poland that differ in terms of their location above sea level and, therefore, may influence the proportions of active compounds in berries. Fruits were harvested throughout the entire growth phase (between May and October).

## 2. Results and Discussion

### 2.1. The Composition of A. melanocarpa Fruit Extracts

The content of polyphenols, phenolic acids, and sugars in berries undergo changes during ripening. The acidity and Brix values are generally measured by farmers and used as an indicator of ripeness. Aronia is unique among berries because its fruits ripen and are suitable for harvesting for over one month, which results in a variety of their composition and requires a detailed analysis. The increase in Brix appeared to be due to the increase in sorbitol, representing more than 40% of the total sugar content of Aronia juice [35].

In this study, Aronia berry samples were collected between May (green, unripe) and October (red, overripe) on two eco-friendly plantations in Poland (Table 1).

**Figure 1:**
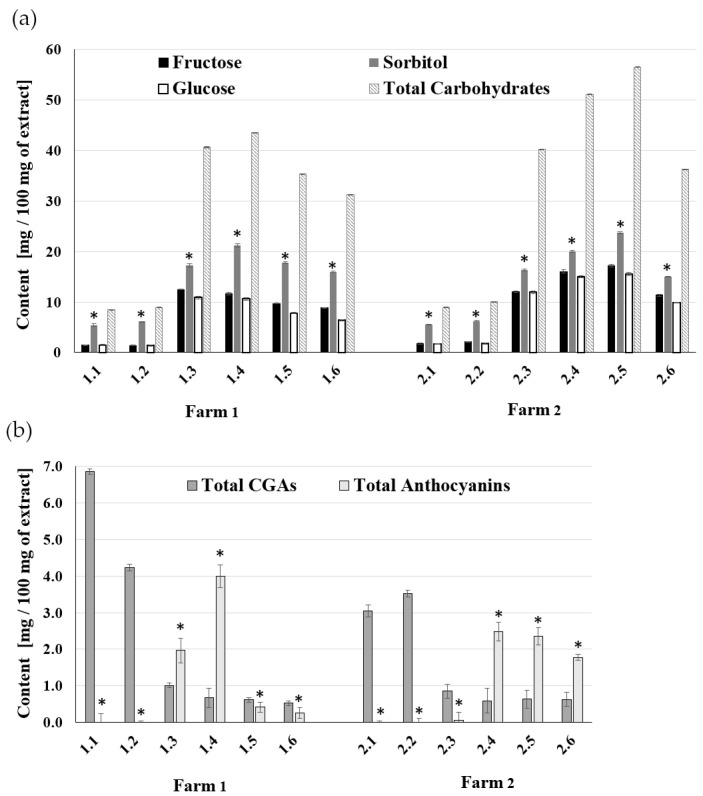
(**a**) The sorbitol, fructose, glucose, and total carbohydrates content in Aronia extracts collected from May to October. (**b**) The changes of chlorogenic acids (CGAs, sum of CGA and nCGA) and anthocyanins (Cya-3-Gal and Cya-3-Ara) content in the same time range. Error bars indicate standard deviation. Statistical significance, * <0.05 by one-way ANOVA with Tukey’s post-test.

The content of chlorogenic acids (CGAs) and anthocyanins in Aronia berry extracts was studied in our previous work [36]. It was shown that there are no anthocyanins but large amounts of chlorogenic acids at the beginning of fruit growth. Later in growth, the fruit turns red and anthocyanins appear, while the CGAs content per fruit weight decreases. These changes (Figure 1b) are a good reference point for research on anti-inflammatory properties, allowing the identification of extracts with the most optimal composition. The content of carbohydrates in the extracts was determined to complete the composition data. HPLC-RI analysis showed the presence of sorbitol, glucose, and fructose (Figure 1b). Sucrose was not detected in the samples, which is in line with data from other works [7]. Compared to other fruits, *A. melanocarpa* berries contain a large amount of sorbitol. A relatively small amount of sorbitol was detected in green fruits. Still, its content began to increase markedly with the ripening of the fruit and the appearance of anthocyanin pigment and then decreased slightly towards the end of the ripening period (Figure 1a). The time related to the ripening season at Farms 1 and 2 was also noticeable. For fruits from the region with a mild climate (Farm 1), the maximum sorbitol content occurred in August (samples 1.4), while in the higher elevation region with a delayed growing season (Farm 2) the maximum occurred in September (samples 2.5). At the given time, the fruits of Farm 2 contained a slightly higher amount of sorbitol—24 mg/100 mg of extracts—compared to the 21 mg/100 mg of Farm 1. The maximum content of total carbohydrates (sorbitol + fructose + glucose) in extracts 1.4 was 44% for Farm 1 and 57% for Farm 2 samples 2.5. Similar tendencies were observed for glucose and fructose, including a shift in a vegetation period. The fruits of Farm 2 contained a slightly higher amount of these two sugars (17 mg/100 mg of Fru and 16 mg/100 mg for Glu) compared to the extracts of Farm 1 (12 mg/100 mg of Fru and 11 mg/100 mg of Glu).

The observed trends in the content of the carbohydrate composition are consistent with the results obtained for anthocyanins (Figure 1b). However, the total anthocyanin content in sample 1.4 from Farm 1 was 4.0 mg/100 mg and was higher than in sample 2.4 from Farm 2 with the higher altitude (2.5 mg/100 mg extract).

A variable content of chlorogenic acids (CGAs) was observed depending on the stages of plant vegetation. Green chokeberry fruit, containing large amounts of these acids (chlorogenic and neochlorogenic), turned out to be a valuable raw material (Figure 1b, 1.1; 1.2 and 2.1; 2.2). These acids are beneficial to Aronia preparations due to their blood glucose-lowering effect and antimicrobial activity. The levels of anthocyanins and chlorogenic acids in berries undergo specific changes during the ripening process and depend on harvest timing and cultivation region. Additionally, a correlation has been observed between the reduction in CGAs over time and the emergence of anthocyanins. Our results showed that low-altitude, mild-climate cultivation favored fruits with higher levels of chlorogenic acids. In addition, green fruits, rich in chlorogenic acids and with very low sugar content, are valuable raw materials.

### 2.2. Anti-Atherosclerotic Activity in Human Endothelial Cells

**Figure 2:**
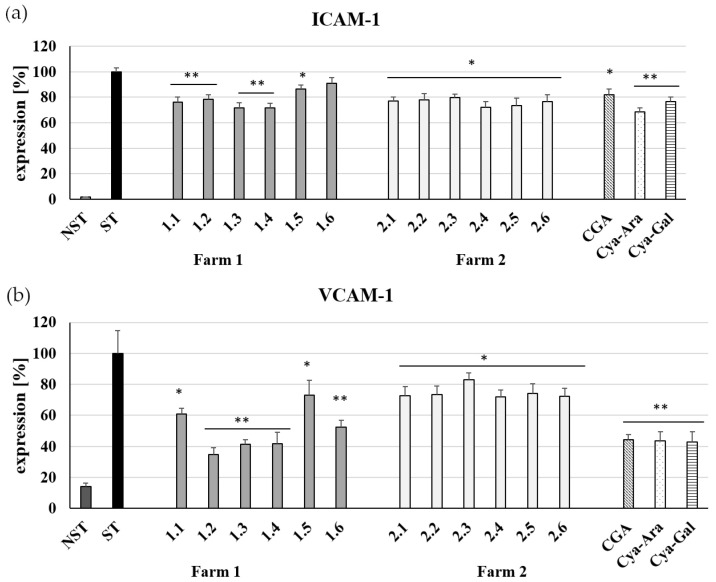
(**a**) The influence of *A. melanocarpa* extracts from Farm 1 and Farm 2 on ICAM-1 expression. (**b**) VCAM-1 expression by TNF-α stimulated HUVECs (ST). CGA, Cya-Ara, and Cya-Gal at concentrations of 50 μM were used as positive controls. Data from three separate experiments assayed in triplicate are expressed as mean ± (SD). Statistical significance, * <0.05; ** <0.005 versus stimulated control (Dunnett’s post hoc test); ST, stimulated control; NST, non-stimulated control.

As inflammation and cell adhesion molecules located on endothelial cells cause leucocyte adhesion to the endothelium, we investigated the effects of *A. melanocarpa* fruit extracts on TNF-α induced ICAM-1 and VCAM-1 expression by human umbilical vein endothelial cells (HUVECs). Flow cytometry results showed that all Aronia berry extracts collected from May to October from both farms inhibit TNF-α stimulated ICAM-1 and VCAM-1 molecules. This is illustrated in Figure 2.

Extracts containing mainly chlorogenic acid, i.e., without anthocyanins (no 1.1 and 1.2), suppressed the surface expression of the TNF-α-stimulated ICAM-1 by 1.31-fold and 1.27-fold, respectively (Figure 2a, 1.1; 1.2) and inhibited surface expression of the TNF-α-stimulated VCAM-1 molecule by 1.6-fold and 2.4-fold, respectively (Figure 2b, 1.1; 1.2). However, as the content of anthocyanins in berry extracts increases, its anti-inflammatory effect also increases. This is best seen in the case of extracts 1.3 and 1.4 from July and August, as illustrated in Figure 2. These extracts decreased TNF-α-stimulated ICAM-1 surface expression by 1.4-fold (Figure 2a, 1.3;1.4), while VCAM-1 was lowered by almost 2.5 times (Figure 2b, 1.3; 1.4).

The *A. melanocarpa* berries from Farm 2 were also collected from May to October. This farm is at a higher elevation, where plant vegetation starts later than in central Poland. All berry extracts from this plantation inhibit TNF-α-stimulated ICAM-1 and VCAM-1 molecules. This is illustrated in Figure 2. We have found that green berry extracts containing mainly chlorogenic acid, i.e., 2.1 and 2.2, suppressed TNFα-stimulated ICAM-1 by 1.30-fold and 1.28-fold, respectively (Figure 2a). Regarding the surface expression of the TNF-α-stimulated VCAM-1 molecule, extracts No. 2.1 and 2.2 inhibited it at the same level, that is, by 1.3 times (Figure 2b). As the anthocyanin content in the chokeberry extracts increases during fruit ripening, the chlorogenic acid decreases. This is best seen in extract No. 2.4 from August, as illustrated in Figure 1. This extract reduces the surface expression of TNF-α-stimulated ICAM-1 and VCAM-1 at a similar level, i.e., 1.4 times (Figure 2a, 2.4) and 1.3 times, respectively (Figure 2b, 2.4). The results of surface expression levels of ICAM-1 and VCAM-1 for Aronia fruit extracts were compared with the effects of pure reference compounds, i.e., chlorogenic acid, cyanidin 3-arabinoside, and cyanidin 3-galactoside.

### 2.3. Influence on ICAM-1, VCAM-1, IL-6, and MCP-1 mRNA Expression

**Figure 3:**
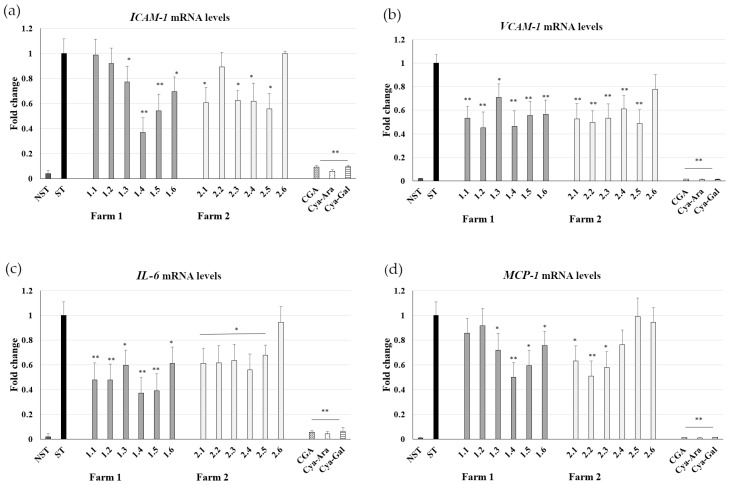
The influence of *Aronia melanocarpa* extracts from Farm 1 and Farm 2 on (**a**) ICAM-1 expression, (**b**) VCAM-1, (**c**) IL-6, and (**d**) MCP-1 expression by TNF-α stimulated HUVECs (ST). CGA, Cya-3-Ara, and Cya-3-Gal at concentrations of 50 μM were used as positive controls. Data from three separate experiments assayed in triplicate are expressed as mean ± SD. Statistical significance, * <0.05; ** <0.005 versus stimulated control (Dunnett’s post hoc test); ST, stimulated control; NST, non-stimulated control.

The effect of chokeberry fruit extracts on *VCAM-1* and *ICAM-1* mRNA expression levels was studied in TNF-α-stimulated HUVECs. TNF-α stimulation significantly increased *VCAM-1* and *ICAM-1* mRNA levels in HUVECs. HUVECs pretreated with 50 µg/mL of Aronia extracts no. 1.2 and 1.4 (Farm 1) showed a *VCAM-1* mRNA expression level comparable to that of nonstimulated cells (Figure 3b). As shown in Figure 3a, extract 1.4 also decreases markedly (by 2.7-fold) in TNF-α stimulated *ICAM-1.* The level of inhibition of mRNA appeared to be comparable with the level of inhibition of surface protein expression. The quantitative reverse transcription polymerase chain reaction in real-time revealed that extract 2.5 from berries collected in August at Farm 2 most effectively repressed *VCAM-1* (by 2-fold; Figure 3b) and *ICAM-1* (by 1.8-fold; Figure 3a) genes.

Endothelial cell dysfunction involves the induction of genes encoding leukocyte adhesion molecules (e.g., VCAM-1 and ICAM-1), chemotactic factors (e.g., monocyte chemoattractant protein-1; MCP-1), and cytokines (e.g., IL-6). Thus, the effect of *A. melanocarpa* extracts from Farm 1 and Farm 2 on *IL-6* and *MCP-1* mRNA expression in stimulated endothelial cells was assessed. HUVECs pretreated with 50 µg/mL of Aronia extract no. 1.4 showed a 2.68-fold lower level of *IL-6* mRNA expression compared to nonstimulated cells (Figure 3c). As shown in Figure 3d, extract 1.4 decreases *MCP-1* mRNA by 2.0-fold in TNF-α stimulated HUVECs. On Farm 2, extract no. 2.2 (Figure 3d) inhibited *MCP-1* similarly strongly, reflecting its high content of CGAs and low carbohydrate content.

Inflammatory mediators, oxidants, and abnormal blood flow cause vascular endothelium damage. Alterations in endothelial cells are considered the key factor in the pathogenesis of atherosclerosis, which is the primary initiator of cardiovascular disease. Overexpression of endothelial adhesion molecules, including intercellular adhesion molecule 1 (ICAM-1) and vascular cell adhesion molecule-1 (VCAM-1), play a critical role in the dysfunction of the vascular endothelium and mediate leukocyte recruitment. The accumulation of monocytes and lymphocytes accompanied by the expression of VCAM-1 and ICAM-1 on the surface of the endothelium of the aorta was shown, and the number of VCAM-1 positive cells was reported to increase, especially in advanced lesions, and to play a role in both lesion formation and expansion [37]. The intercellular adhesion molecule (ICAM)-1 is also present in atherosclerotic lesions and plays a role in their progression [38]. The upregulation of endothelial adhesion molecules is increased by inflammatory mediators such as IL-1, TNF-α, and lipopolysaccharide (LPS) [39,40]. The present study also showed that HUVEC treatment with TNF-α upregulated the expression of ICAM-1 and VICAM-1. HUVECs pretreated with extracts 1.2 and 1.4 (June and August) showed a two-time lower level of TNF-α induced *VCAM-1* mRNA expression compared to the control. Additionally, treatment with extract 1.4 was associated with a significantly lower *ICAM-1* mRNA expression level. To our knowledge, the present study is the first to report that, depending on the fruit harvest time, the extracts express different inhibitory effects on the gene and protein expression of ICAM-1 and VCAM-1, the predominant endothelial adhesion molecules. Furthermore, *A. melanocarpa* extract 1.4 markedly decreased the expression of *MCP-1* and *IL-6* mRNA expression in TNF-α stimulated HUVECs. The anti-inflammatory effects of Aronia berry extracts were previously ascribed to cyanidin-3-arabinoside and quercetin [41]. However, these polyphenols are present in very small amounts in berry extracts. Our results suggest that the studied berry extracts exert anti-atherosclerotic effects and inhibit *VCAM-1* and *ICAM-1* gene expression due to particular bioactive compounds, i.e., chlorogenic acids and anthocyanins. A limitation of our study is that we cannot exclude a potential synergistic effect with other compounds, such as proanthocyanidins and flavonols found in chokeberry berries. At the beginning of the ripening process of chokeberry fruit, large amounts of CGAs are observed. Anthocyanins appear later, which coincides with a significant decrease in the content of CGAs. Hence, there is no specific stage during which both these compounds are present in high amounts; thus, their synergistic effect cannot be confirmed. We can only demonstrate the content of chlorogenic acids and anthocyanins related to the berry ripening stage and associated anti-atherosclerotic properties of studied *A. melanocarpa* extracts. Chokeberry is unique among other berries due to its high content of polyphenolic bioactive compounds. According to a study by Koponen et al. [42], a relatively high amount of anthocyanins was also found in blueberry, crowberry, bilberry, and black currant. These fruits are also known to have anti-inflammatory properties, may reduce susceptibility to oxidative stress, and provide antioxidant protection. Gasparrini et al. [43] conducted a review on more than 20 fruits and found that all of them have a significant effect in terms of reducing inflammation caused by LPS. However, it is challenging to determine the fruits with the best anti-inflammatory properties due to various factors that can influence the results, such as extract preparation and their composition, different in vitro and in vivo testing models, and the type of studied inflammatory markers.

Cardiovascular diseases (CVDs) are the leading causes of morbidity and mortality worldwide [44]. Atherosclerosis is the main CVD risk factor. Blocking agents targeting endothelial adhesion molecules, including the intercellular adhesion molecule-1 (ICAM-1) and the vascular cell adhesion molecule-1 (VCAM-1), have been proposed to prevent atherosclerosis [45,46,47]. The experimental data obtained from in vivo and in vitro studies have shown that medicinal herbs may also have therapeutic value in CVD [48]. Thus, the positive effect of *A. melanocarpa* on atherosclerosis and its underlying mechanism deserves further study. In this study, some of the extracts show an anti-atherosclerotic and anti-inflammatory nature by inhibiting surface expression of adhesion molecules and gene expression of inflammatory mediators in endothelial cells. ICAM-1 and VCAM-1 expression was significantly suppressed by extracts rich in chlorogenic acids (from green fruits) and anthocyanins (from ripe berries). The anti-inflammatory activities of the berry extracts depend on the time of harvest (August–September) and the cultivation region. Furthermore, our results indicate that chokeberry fruits at a very early maturation stage have therapeutic potential in preventing atherosclerosis and other inflammatory vascular diseases.

### 2.4. PCA Analysis

The results were analyzed using chemometric methods and multivariate statistics (PCA). The first three principal components explain more than 90% of the sample variation. The analysis of the two variables, PC1 and PC2, provides valuable information.

**Figure 4:**
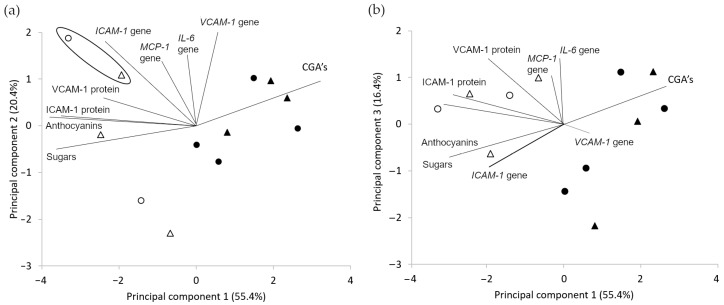
Score plot of PC1 vs. PC2 (**a**) and PC1 vs. PC3 (**b**) for fruits from both farms. Farm 1 ripe fruits (⚪), Farm 1 other fruits (⚫), Farm 2 ripe fruits (△), Farm 2 other fruits (▲). On the plot, (**a**) the best fruits from Farm 1 and Farm 2 are marked.

The PC1 variable is correlated with fruit ripening. There is a separation of unripe (rich in chlorogenic acids) and ripe (rich in anthocyanins) fruits along the variable PC1 (Figure 4). On both farms, the unripe fruits (1.1, 1.2, 2.1, 2.2) correspond to the points in the first quadrant. They correlate with the highest content of chlorogenic acids. On the other side of the *Y*-axis, the points correspond to mature fruit, correlating with the content of anthocyanins, sugars, VCAM-1 and ICAM-1 protein levels, and the *ICAM-1* gene expression (1.3, 1.4, 2.4, 2.5, 2.6). At the same time, using the variables PC1 and PC2, it is possible to indicate among them the best extracts selected based on the berry samples collected for analysis from two farms with specific geographical origins presented in Table 1. In Figure 4a, points 1.4 and 2.5, significantly different from the others, are marked. Among ripe fruits, those with the most potent biological properties (the highest decrease in the expression of *ICAM-1* and *VCAM-1* genes) are separated along the variable PC2. The remaining ripe fruits (1.3, 2.4, and 2.6) correspond to the points in the third quadrant. They have weaker biological properties but a relatively high content of anthocyanins and sugars. There are overripe fruits (1.5, 1.6) in the fourth quadrant of Farm 1 for which the content of anthocyanins has decreased, and the expression of the protein and gene of VCAM-1 and ICAM-1 has also decreased. For Farm 2 in the fourth quadrant, there is a point corresponding to fruits 2.3 that are still unripe, with increased content of anthocyanins, but lower than for 2.4, 2.5, and 2.6.

Phenolic compounds have the most significant influence on the expression of *IL-6* and *MCP-1*. Both high concentrations of anthocyanins and CGA result in higher inhibition of *MCP-1* and *IL-6* expression. Earlier observations showed [49,50] that these properties are not correlated with one group of polyphenolic compounds, but the sum of these compounds has been confirmed. PCA allowed us to notice that the content of CGAs and anthocyanins in fruits from the two farms differs not only in the period of occurrence but also in the amount. Hence, the best results of the inhibition of *MCP-1* and *IL-6* gene expression were obtained for fruits 1.4, where the content of polyphenolic compounds was the highest.

The PCA analysis clearly shows the differences between the two farms. For Farm 1, the stages of unripe (1st quadrant), ripe (2nd and 3rd quadrant), and overripe (4th quadrant) fruit can be distinguished. Farm 2 in the 1st quarters are unripe fruits, in the 4th is a fruit during ripening, and in the 2nd and 3rd are ripe fruits. Fruit ripening is delayed on Farm 2 compared to Farm 1. Furthermore, it can be seen that, after ripening, the fruit of Farm 2 maintains a high anthocyanin content longer but does not exceed the biological activity of fruit from Farm 1 (1.4).

## 3. Materials and Methods

### 3.1. Plant Material and Extract Preparation

*Aronia melanocarpa* (Michx.) Ell. samples of berries were randomly harvested from two eco-friendly commercial farms in 2016 between May and October (Table 1). Farm 1 is located in central Poland, with a moderate climate, classified as Cfb (Köppen scale), and the average altitude is approximately 90 m above sea level. Farm 2 is located in southwestern Poland, with an average altitude of ca. 450 m ASL, with a slightly harsher semi-continence climate, on the border of climatic zones Cfb and Dfb (Köppen scale) [51]. The plants on both farms came from a specialized organic chokeberry nursery in Grójec, Poland. The plant material was identified by Piotr Eggert (M.Sc.). A voucher specimen (no 10192) was deposited in the herbarium of the Department of Biology and Pharmaceutical Botany (GDMA), Medical University of Gdansk, and is available upon request.

Berry samples were frozen immediately after harvesting, lyophilized (Alpha 1-2 LD plus Martin Christ), ground into a powder, and frozen until analysis. Next, the three portions of each sample (described by date of collection, 1 g each) were extracted with methanol acidified with 1M HCl. The extraction was performed by sonication for 20 min at room temperature; then, the samples were centrifuged. Supernatants were collected, evaporated to dryness, and stored at −30 °C. Fresh extract solutions were prepared before each analysis.

### 3.2. Content of Anthocyanins and Chlorogenic Acids in A. melanocarpa Extracts 

Extracts were analyzed for the content of anthocyanins, chlorogenic acids, and carbohydrates (sugars and sugar alcohol-sorbitol).

Our previous publication described in detail the method of determining chlorogenic acids and anthocyanins in extracts, including the high-performance liquid chromatography coupled with diode array detector (HPLC-DAD) procedure with validation [36]. Based on these results, six extracts from each farm were selected for biological research (Table 1), showing changes in the content of anthocyanins and CGAs over time.

### 3.3. Determination of Sorbitol and Sugars in Extracts of A. melanocarpa by HPLC-RI

**Figure 5:**
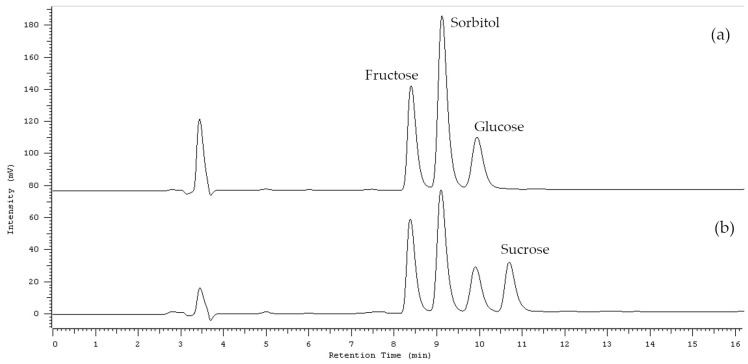
HPLC-RI chromatograms of *A. melanocarpa* extract (**a**) and standards with sucrose (**b**).

All extracts have been tested for fructose, sucrose, glucose, and sorbitol content using high-performance liquid chromatography coupled with a refractive index detector (HPLC-RI) method. Qualitative and quantitative analysis was performed by HPLC using the Hitachi Chromaster HPLC system (Hitachi High-Technologies Corporation, Tokyo, Japan) equipped with a gradient pump, a refractive index detector, a column oven, and an autosampler. Chromatographic separations were carried out on a Merck Purospher STAR NH_2_ column (5 μm, 250 mm × 4.6 mm) at 35 °C, under isocratic conditions, mobile phase: acetonitrile/water 75/25 (*v*/*v*), a flow rate of 1 mL/min. All measurements were made in triplicate. Concentrations were determined using sorbitol, glucose, and fructose standards calibration curves. No sucrose was detected in the samples (Figure 5). The linearity of the method was assessed by examining the coefficient of determination (R^2^) from the calibration curve obtained for each standard. No significant difference was observed for the chromatograms of the standard and experimental solutions.

The limit of detection (LOD) and limit of quantification (LOQ) were determined using the following formulas: LOD = 3.3 × σ/S and LOQ = 10 × σ/S, where σ represents the standard deviation of the y-intercept, and S corresponds to the slope of the calibration curve (as shown in Table 2). Furthermore, the method’s accuracy analysis yielded promising results. The % RSD (relative standard deviation) for intraday variation remained consistently low, measuring less than 2%. The recovery results fell within an acceptable range of 97% to 103% across different days, indicating that the method is accurate.

### 3.4. Anti-Atherosclerotic Activity in Human Endothelial Cells 

#### 3.4.1. Materials

Human umbilical vein endothelial cells (HUVECs), EBM-2 (Basal endothelial medium), EGM-2 Bulletkit (EBM-2 + all growth supplements), Hanks balanced salt solution (HBSS), fetal bovine serum (FBS), trypsin-EDTA (ethylenediaminetetraacetate), and trypsin neutralizing solution (TNS) were obtained from LONZA (Basel, Switzerland). 3-(4,5-dimethylthiazol-2yl)-2,5-diphenyltetrazolium bromide (MTT), dimethyl sulfoxide (DMSO), and non-enzymatic cell dissociation solution were provided by Sigma (St. Louis, MO, USA). Human recombinant TNF-α, phycoerythrin (PE)-conjugated mouse monoclonal antibody conjugated to phycoerythrin (PE), mouse monoclonal antibody conjugated to fluorescein isothiocyanate (FITC), mouse anti-human VCAM-1 monoclonal antibody conjugated to VCAM-1, and suitable conjugated mouse IgG isotypes were purchased from Becton Dickinson (San Diego, CA, USA). All other chemicals were purchased from Sigma-Aldrich (St. Louis, MO, USA).

Before use, the extracts were dissolved in PBS buffer containing 10% DMSO and were diluted with a culture medium. All reagents were maintained at −20 °C, and dilutions were made with a culture medium.

#### 3.4.2. Cell Culture and Experimental Conditions

HUVECs were cultured in an endothelial cell growth medium with 2% FBS. Cells were maintained at 37 °C in a 5% CO_2_ in a humidified atmosphere and were used for experiments between passages 3 and 4 (doubling population < 10). Human umbilical vein endothelial cells were cultured in 6- or 12-well plates. When HUVECs reached confluence, they were pretreated with *A. melanocarpa* extracts (50 μg/mL) and then treated with TNF-α (10 ng/mL) for the indicated period of time.

#### 3.4.3. Cell Viability Assessment by MTT Assay

Cell viability was assessed by determination of the conversion of the MTT salt [3-(4,5-dimethylthiazol-2-yl)-2,5-diphenyltetrazolium bromide] by mitochondrial dehydrogenase. Briefly, cells were incubated for 24 h in 24-well plates at a concentration of 50 μg/mL of the tested extracts and for another 4 h with 0.5 mg/mL of MTT, which is converted in living cells by the mitochondrial dehydrogenase to insoluble formazan. The converted dyes were then dissolved in 0.04 M HCl in absolute isopropanol. The absorbance was measured spectrophotometrically at 570 nm using an Epoch microplate reader (BioTek Inc., Winooski, VT, USA) equipped with Gen5 software (version 2.01, BioTech Instruments, Inc., Biokom, Winooski, VT, USA). Cell viability remained constant in all experiments (more than 90%).

#### 3.4.4. Measurement of ICAM-1 and VCAM-1 Expression in Human Umbilical Vein Endothelial Cells by Flow Cytometry

HUVECs planted in 12-well plates were pretreated with 50 μg/mL of Aronia extracts for 2 h and then treated with TNF-α (10 ng/mL) for 16 h. Then, the cells were harvested and washed in phosphate-buffered saline solution (PBS) containing 1% FBS and resuspended in 100 μL of labeling buffer. Immediately after that, cells were incubated with PE-conjugated mouse anti-human ICAM-1 and FITC-conjugated mouse anti-human VCAM-1 antibodies. This incubation was conducted in a dark place and lasted 1 h. For the isotype control, cells were treated with PE-conjugated mouse anti-IgG1 antibody. The samples were rewashed with PBS and analyzed using a FACSCalibur flow cytometer (BD, Biosciences, Franklin Lakes, NJ, USA) using CellQuest Pro software version 3.1. After correcting for nonspecific binding using the isotype control, the mean fluorescence intensity (MFI) was assessed as an indicator of ICAM-1 and VCAM-1 surface protein expression.

#### 3.4.5. Determination of Gene Expression

RNA was extracted using the Total RNA Mini kit (A&A Biotechnology, Gdynia, Poland) and treated with DNase (DNA-free™, Ambion, Austin, TX, USA). RNA concentration and purity were measured with a microvolume UV-vis spectrophotometer (Quawell Q3000, Quawell Technology Inc., Sunnyvale, CA, USA). RNA was reverse transcribed to cDNA with a high-capacity RNA-to-cDNA kit (Applied Biosystems, Waltham, MA, USA). The expression levels of the *VCAM-1*, *ICAM-1*, *IL-6*, and *MCP-1* genes were assessed with the quantitative real-time PCR (qRT-PCR) method. The following TaqMan gene expression assays (Thermo Fisher Scientific, Inc., Waltham, MA, USA) were used; ICAM1 assay ID: Hs00164932_m1, VCAM1 assay ID: Hs01003372_m1, IL-6 assay ID: Hs00985639_m1, MCP-1 assay ID: Hs00234140_m1. Analyses were performed on a ViiA 7 system (Applied Biosystems; Thermo Fisher Scientific, Inc.). Data were normalized to the reference genes (GAPDH-Hs99999905_m1, B2M-Hs99999907_m1), and relative quantification of the messenger RNA levels was performed using the 2^ΔΔCT^ method [52]. All qRT-PCR experiments were performed in triplicate. A mean value was used for the determination of studied mRNA levels.

### 3.5. PCA Analysis 

PCA analysis was performed using Statistica™ 13.3 software (TIBCO Software Inc., Palo Alto, CA, USA). All data used for PCA—sugar content, anthocyanins, chlorogenic acids, expression of the genes ICAM-1, VCAM-1, MCP-1, and IL-6, and protein ICAM and VCAM expressed as folds—were first normalized using Z-score normalization.

### 3.6. Statistical Analysis

The results were expressed as mean ± SD; the error bars represent the range of analytical replicates, *n* = 3. Data were evaluated using Statistica™ 13.3 software (TIBCO Software Inc., Palo Alto, CA, USA). One-way analysis of variance (ANOVA) followed by Tukey’s or Dunkan’s tests was performed to test significance at *p* < 0.05.

## 4. Conclusions

In this study, the anti-atherosclerotic properties of *Aronia melanocarpa* extracts influenced by their chemical composition associated with the ripening stage of the berries were reported. The novelty of our research was the study of green fruits of *A. melanocarpa,* a potential raw material for functional food. In this study, we identified the *Aronia melanocarpa* extracts from fruits harvested from May to October from two locations, which were able to reduce the surface expression of TNFα-stimulated ICAM-1 and VCAM-1 molecules. This effect was also confirmed by the reduction of *ICAM-1* and *VCAM-1* mRNA. The proportions of active compounds in berries and their anti-atherosclerotic properties were associated with the location of the studied plantations. The proposed methodology makes it possible to determine which compounds from phenolic acids (neochlorogenic and chlorogenic) and anthocyanins are associated with a decrease in the level of ICAM-1 and VCAM-1 adhesion molecules in relation to the harvest period. Therefore, it can be concluded that berries have a higher biological activity or maintain a high anthocyanin content longer. These results are even more interesting considering that the content of chlorogenic acids and anthocyanins changes during fruit ripening and depends on the time of harvest and the region of cultivation. In green unripe fruit, there are no anthocyanins, so the anti-atherosclerotic effect of these samples may be associated with a high content of chlorogenic acids. A slightly better anti-inflammatory effect was observed for fruit extracts from a farm located in a temperate climate. This conclusion is based on the results of the lower levels of surface expression of the VCAM-1 molecule and lower levels of *IL-6* mRNA expression. This may be due to a higher chlorogenic acid content in these extracts compared to extracts from farms in a mountainous area with a harsher climate. This suggests that it is not only anthocyanins that have anti-inflammatory effects on endothelial cells. These findings provide new information suggesting that even unripe berries, rich in phenolic compounds, may have therapeutic potential in preventing atherosclerosis and other inflammatory diseases.

## Figures and Tables

**Table 1:** Numbering of chokeberry samples collected from two farms.

Date of Collection	Farm 1	Farm 2	Ripening/Color
26 May	1.1	2.1	unripe/green
26 June	1.2	2.2	
24 July	1.3	2.3	ripe/dark red
21 August	1.4	2.4	
18 September	1.5	2.5	
30 October	1.6	2.6	overripe/black

**Table 2:** Concentration range, limit of detection (LOD), and limit of quantification (LOQ) for sorbitol and sugars by HPLC-RI.

Compound	RT (min)	Calibration Curve	R^2^	Linear Range (mg/mL)	LOD (mg/mL)	LOQ (mg/mL)
Sorbitol	8.76	A = 348,714c − 8658	1.000	0.50–12.0	0.06	0.18
Fructose	8.02	A = 342,274c − 7039	0.999	0.30–10.0	0.08	0.25
Glucose	9.21	A = 260,169c − 7766	0.999	0.30–10.0	0.09	0.27

A—peak area, c—concentration (mg/mL) of the compound.

## Data Availability

Data is contained within the article.
